# Optimization of Glucose Powered Biofuel Cell Anode Developed by Polyaniline-Silver as Electron Transfer Enhancer and Ferritin as Biocompatible Redox Mediator

**DOI:** 10.1038/s41598-017-12708-6

**Published:** 2017-10-05

**Authors:** Sufia ul Haque, Inamuddin, Abu Nasar, B. Rajender, Anish Khan, Abdullah M. Asiri, Ghulam Md Ashraf

**Affiliations:** 1 Advanced Functional Materials Laboratory, Department of Applied Chemistry, Faculty of Engineering and Technology, Aligarh Muslim University, Aligarh, 202002 India; 2 Chemistry Department, Faculty of Science, King Abdulaziz University, Jeddah, 21589 Saudi Arabia; 3 Centre of Excellence for Advanced Materials Research, King Abdulaziz University, Jeddah, 21589 Saudi Arabia; 4 King Fahd Medical Research Center, King Abdulaziz University, Jeddah, Saudi Arabia

## Abstract

Polyaniline-silver (PANI-Ag)/ferritin (Frt)/glucose oxidase (GOx) biocompatible anode was utilized for creating power from glucose. The synthesized nanocomposite was investigated by EIS (Electrochemical impedance spectroscopy), XRD (X-ray diffraction), FTIR (Fourier transform infrared spectroscopy), SEM (Scanning electron microscopy), CV (Cyclic voltammetry), and LSV (Linear sweep voltammetry) to know the morphology, crystallinity and electrochemical behaviour of the nanocomposite. The electroactive support (PANI-Ag) was utilized for the immobilization of the enzyme (GOx) and a biocompatible mediator (Frt) to enhance the electrical signals. The electrochemical estimations of the manufactured bioanode were done by utilizing cyclic voltammetry (CV) and linear sweep voltammetry (LSV). The current density obtained by the PANI-Ag/Frt/GOx bioanode was observed to be 25.40 ± 2 mA cm^−2^ at 40 mM of glucose concentration at a scan rate of 100 mVs^−1^.

## Introduction

The global energy demand is increasing every year. Though the petroleum products are presently meeting much of this demand, the problem is its sustained supply and pollution which are serving as the main impetus for research into alternative renewable energy technologies^1,2^. Biofuel cell (BFC) utilizes biological moieties such as enzymes and microbes to directly generate power from the chemical energy contained within various biological matters^3^. Enzymatic biofuel cell (EFC) utilizes enzyme to catalyze the electron flow from substrate like glucose rather than precious metal, in short, they run on sugar^4^. However, the EFCs are more even emerging technology and yet to be widely used. In a recent study, researchers find that the EFC can run on the glucose of the body and the results are really surprising^5^. They can be incredibly used for any kind of biological implant as they run on glucose and enzymes already present in the body that never needs changing or charging^2^. EFC has few downsides, such as the power generation is relatively low, the mechanism is complicated and finally, it’s hard to strip an electron from an enzyme than a precious metal^6^. However, the clean energy obtained from EFC is quite perfect to power a biological implant. EFC can prove to be very useful, probably will see them in future with their complete utilization. The basic requirements for EFC are biocompatibility, long-term stability, integration into biomedical devices and sufficient power output. But the current issues associated with EFCs are their short life span and poor power density.

Enzymatic biofuel cell is supposed to have a good ability to power a micro-scale electronic and biomedical devices^2,7–9^. Redox enzymes are the primary catalysts used to generate the power by initiating the redox reaction. For developing EFC, different redox anodic enzymes are reported so far: glucose oxidase^10,11^, glucose dehydrogenase^9,12,13^, alcohol dehydrogenase^14,15^, aldehyde dehydrogenase^16^ and fructose dehydrogenase^17^ whereas bilirubin oxidase^12^, laccase^18,19^, horseradish peroxidase^20,21^ and microperoxidase^22^ are used at the cathode. For generating the power, the enzyme selection for the fabrication of bioelectrode depended on the choice of the substrate being utilized. The substrate used should be cheap and renewable which do not harm the enzymatic function. Especially for implantable biomedical devices, glucose is considered as an ideal fuel. The performance of biofuel cells basically depends on the selection of anodic and cathodic configuration^23–26^. However, the anodic enzyme glucose oxidase has apoenzyme as an electron transferring unit which is deeply buried inside its structure^27,28^. So the sufficient electrical communication is quite difficult between the enzyme and the electrode, thus to overcome this issue conductive polymers are used^6,29,30^. Polyaniline-Silver (PANI-Ag) is utilized as a conductive material for providing a better communication between redox active site of the enzyme and the surface of electrode^31^. PANI is a commonly used conducting polymer, which has efficient abilities to transfer energy due to its exceptionally porous nanostructure and outstanding electronic properties^32,33^. Furthermore, incorporation of metals like gold, platinum, silver etc., into the polymeric material, has been revealed to be a simple and efficient technique to greatly improve the electrical properties of polymers for realizing a wide range of applications^34–36^. Among all metals, Ag shows the appreciable electrical conductivity^37,38^. A considerable development was ascertained in the electrical conductivities of PANI-Ag nanocomposite compared to those of pure PANI (Emeraldine Base), which increased from 10^−9^ S cm^−1^ to as high as 10^3^ S cm^−1^ ^39–41^. Less work has been done on the doping of PANI with metals that are noble like silver (Ag). The optical, dielectric and electrical properties of PANI can be effectively enhanced by the incorporation of metal (Ag) nanoparticles^40^.

These properties can be changed by the content of metal, size, and shape of incorporating nanoparticles. The highest thermal and electrical conductivities are exhibited by silver among all the metals. Therefore, the composite of Ag with PANI can be a functional composite in terms of high electrical conductivity^41^.

**Figure 1:**
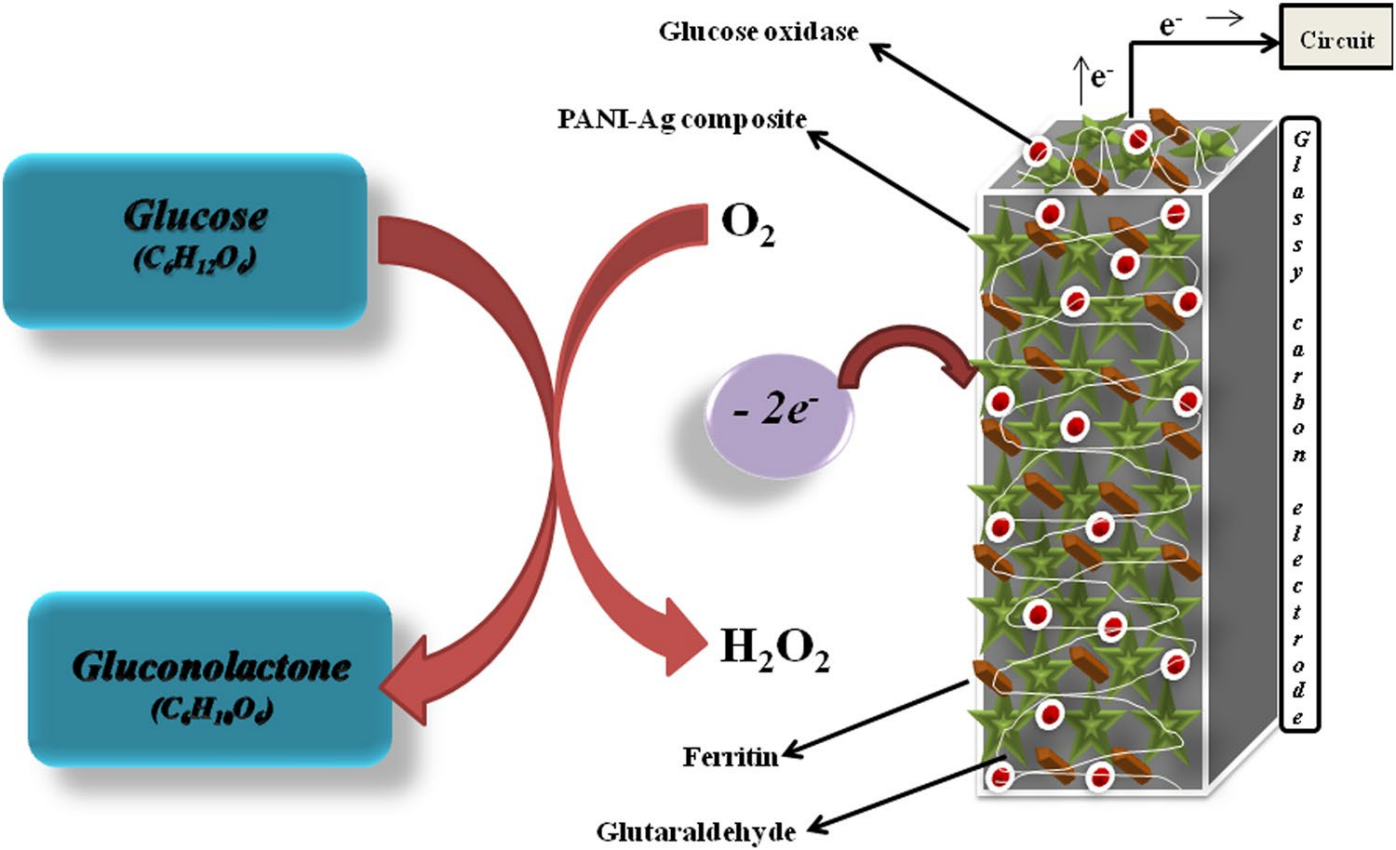
Scheme showing the PANI-Ag/Frt/GOx bioanode and mechanism of glucose oxidation.

This research work is based on the mediated electron transfer, mediator molecule must be biocompatible and eco-friendly so that it can be easily used in biomedical devices that do not harm the patient. Ferritin is such a mediator among the list of non-biocompatible redox mediators holding up to 4500 iron atoms and it also works near to the oxidation potential of glucose oxidase. Thus, this research work was aimed to fabricate the bioanode PANI-Ag/Frt/GOx for biofuel cell assembly as shown in Fig. 1.

## Experimental

### Materials

The ferritin (10 mg mL^−1^ in 0.15 M NaCl) from steed spleen and glutaraldehyde utilized were gotten (Sigma Chemicals, India), phosphate buffer solution (PBS) of pH 5.0 and 7.0 (B2271), (Otto Pvt., Ltd., India), glucose oxidase (Activity 100,000–150,000 units g^−1^ protein) and aniline (Central Drug House, India), nitric acid, silver nitrate and ammonium persulphate (Merck) and D-(+)-glucose anhydrous (Himedia Laboratories Pvt., Ltd., India) were utilized as gotten.

### Synthesis of PANI-Ag nanocomposite

The solution of aniline (0.2 M) was prepared in nitric acid (1 M) and afterward silver nitrate (AgNO_3_) was included as an antecedent. Ammonium persulfate (APS) was utilized as an oxidizing agent to oxidize the above solution and the blend was kept at room temperature. The reaction was slow, carried an induction period of a week. After two weeks green solid polyaniline with Ag particles gel was filtered and was washed with 1 M nitric acid and afterward dried at room temperature^31^.

### Preparation of PANI-Ag nanocomposite dispersion

The PANI-Ag dispersion was made by blending 2 mg of PANI-Ag in 10 mL of dimethylformamide (DMF). The blend was then ultrasonicated for 30 min. The UV–vis spectrophotometer was utilized to check the execution of scattering and ingestion range between 300–700 nm.

### Preparation of PANI-Ag/Frt/GOx electrode

A 0.05 μm alumina slurry was taken to clean the 3 mm diameter of GC (glassy carbon) electrode on a velvet cushion. At that point, the electrode was ultrasonicated for a term of 35 min and washed with distilled water and left to dry at room temperature (25 ± 3 °C). In the wake of drying, 8 μL (optimized) of PANI-Ag dispersion was settled on the GC electrode and is left to dry at room temperature for a term of 3 hours. Further, 4 μL of Frt was dropped on the dried PANI-Ag modified anode and left for 45 min to dry. A 10 mg mL^−1^ of GOx was dissolved in a PBS of pH 5.0 to keep up the activity of the compound while its immobilization is happening. At that point, a 6 μL of GOx was adsorbed on the dried PANI-Ag/Frt modified biocomposite anode and left to dry at room temperature for 60 min. In the end, 6 μL of 2% aqueous solution of glutaraldehyde was drop thrown to cross-interface the PANI-Ag/Frt/GOx bioanode firmly and after that permitted to dry for a time of 40 min. At last, the bioanode was kept in refrigeration until the estimations were taken. The proposed portrayal for the PANI-Ag/Frt/GOx modified electrode is appeared in Fig. 1.

### Characterization

The X- ray diffraction (XRD) of powdered PANI-Ag was recorded using Miniflex TM benchtop XRD framework (Rigaku Corporation, Tokyo, Japan) working at 40 kV and a current of 30 mA with Cu Kα radiation (λ = 1.54 A°). The diffracted intensities were recorded from 20° to 80° 2ϴ points. FT-IR examination of PANI-Ag nanocomposite was recorded by utilizing Nicolet iS50 FT-IR instrument demonstrating absorption spectra in the wavenumber going from 500–4000 cm^−1^ utilizing KBr discs. The surface morphology of the PANI-Ag composite was analyzed by scanning electron microscope instrument (SEM) (JSM6510 LV, JEOL, Japan). All the electrochemical estimations were performed utilizing a PC controlled Potentiostat/Galvanostat (302 N Autolab, Switzerland). A customary three-electrode framework including a working GC electrode (Metrohm 6.1204.300), an Ag/AgCl reference and a platinum wire counter electrodes were utilized for all electrochemical estimation.

The anode was ultrasonicated with advanced ultrasonic cleaner (LMUC arrangement Labman, India).

## Results and Discussion

### XRD study

**Figure 2:**
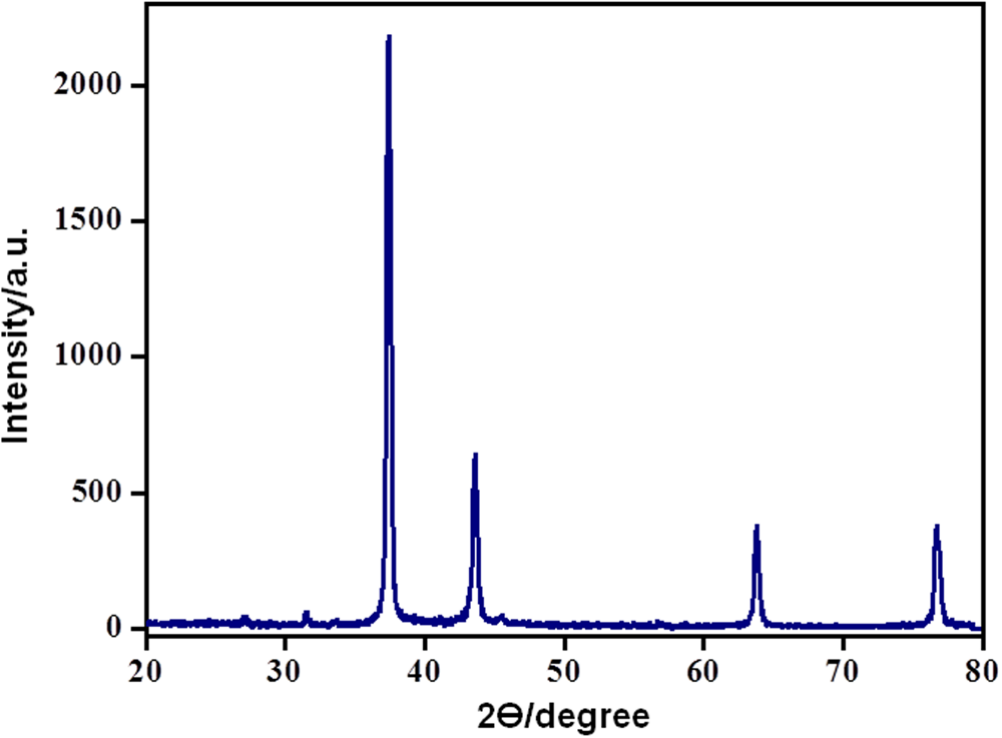
XRD scheme of PANI/Ag nanocomposite.

XRD pattern of PANI-Ag nanocomposites appears in Fig. 2. The PANI-Ag nanocomposite XRD demonstrated and affirms the arrangement of well crystalline Ag nanoparticles. The average crystalline size of the PANI-Ag nanocomposite was figured using Bragg’s reflections at 2θ = 37.2°, 42.4°, 63.8°, and 76.7° which correspond to {111}, {200}, {220}, and {311} lattice planes, respectively, for silver nanoparticles implanted in PANI^31,42,43^. The characteristic peak of PANI-Ag was found at 2θ = 37.2 °. The Scherrer equation was utilized to figure the normal crystalline size which was assessed to be 5.64 nm. The peak widening affirms the development of PANI-Ag nanocomposite^31^.

### FTIR study

**Figure 3:**
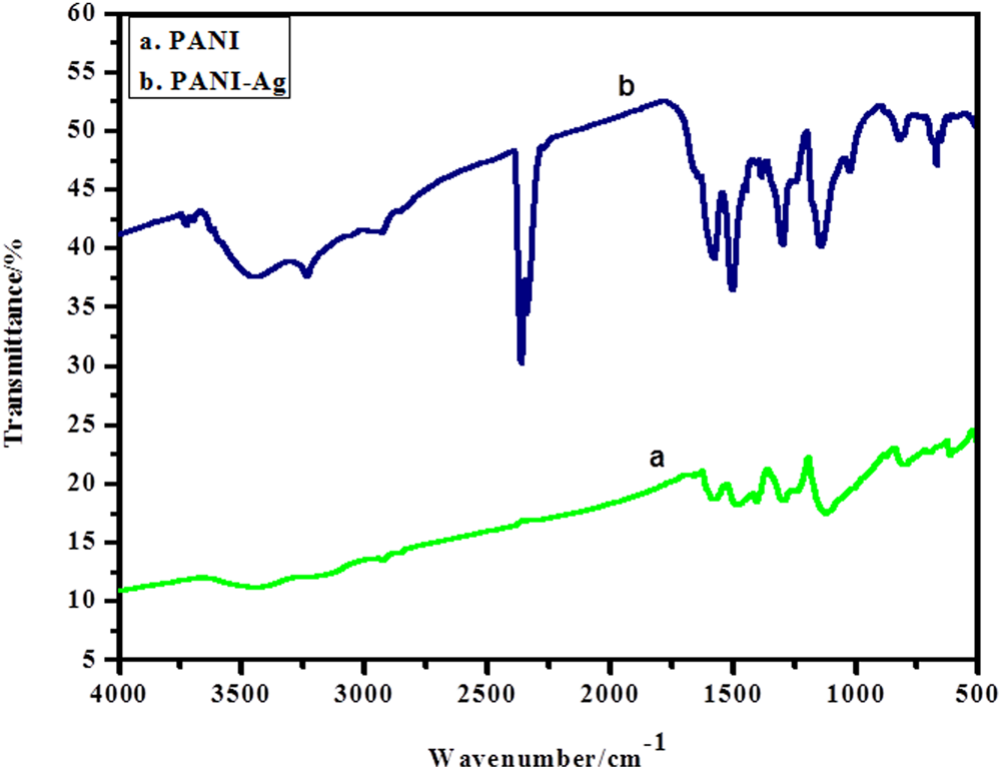
FTIR spectrums of (**a**) PANI, (**b**) PANI/Ag nanocomposite.

Figure 3 demonstrates the FTIR spectrums of PANI and PANI-Ag. The bands at 809.12, 1011.49, 1305.59, 1383.21, 1274.73 and 1496.49 cm^−1^ are corresponding to PANI. The stretching vibrations of benzoid N-B-N and quinonoid N = Q = N structures show up at 1490.24 and 1593.00 cm^−1^, separately. The absorption band at 1256.89 is ascribed to protonation of PANI. The band at 493.21 is because of Ag. The absorption band at 1120.94 cm^−1^ corresponds to PANI in the composite. The vibration method of N = Q = N holding and extending method of the C-N band show up at 1120.94 and 1383.21 cm^−1^, individually^31,43^.

### Morphological study

**Figure 4:**
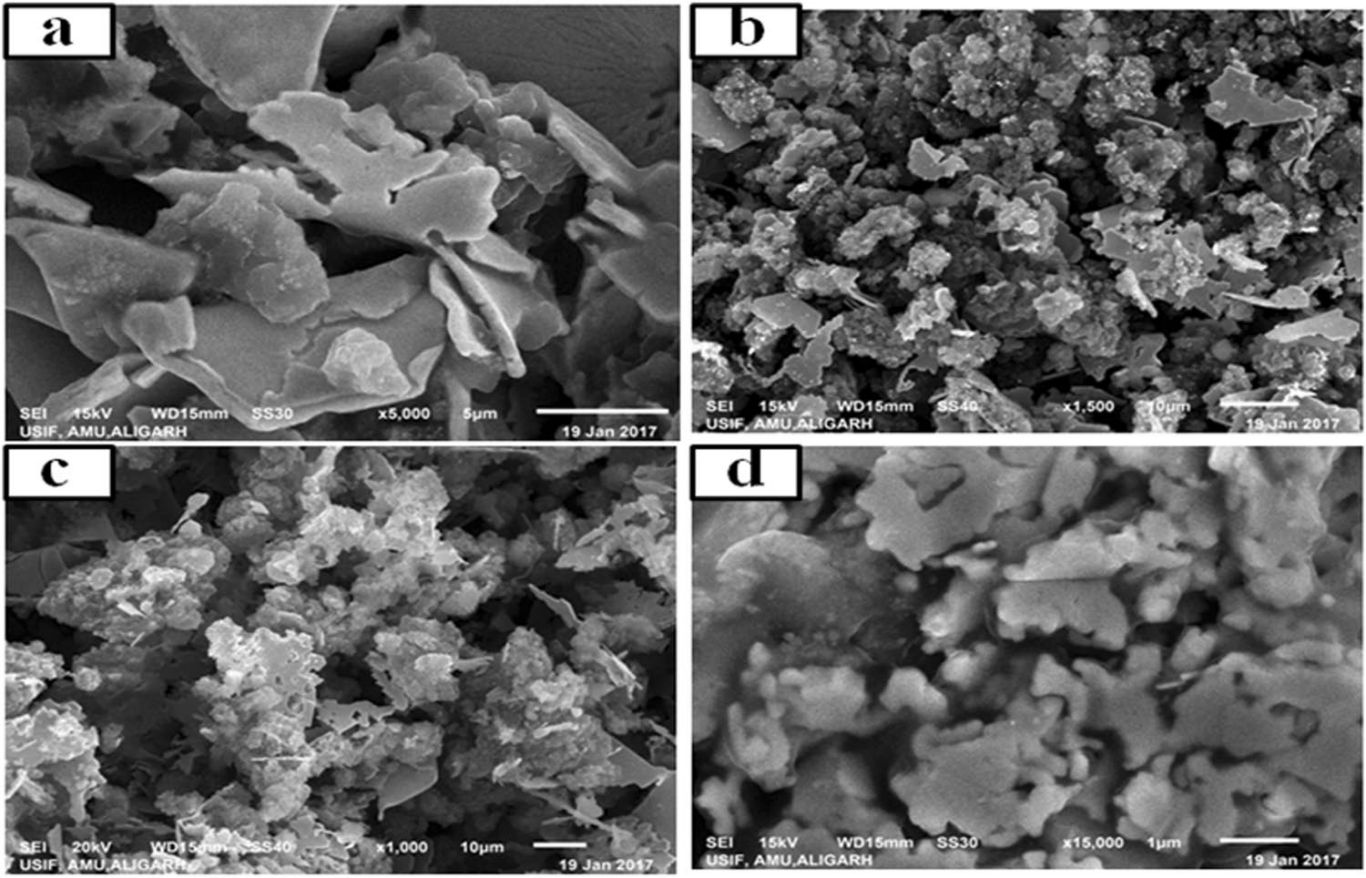
SEM micrographs of (**a**), PANI (**b**), modified PANI-Ag (**c**), modified PANI-Ag/Frt (**d**), modified PANI-Ag/Frt/GOx bioanode.

Figure 4 indicates scanning electron microscopy (SEM) pictures of the (a) PANI, (b) PANI-Ag, (c) PANI-Ag/Frt and (d) PANI-Ag/Frt/GOx. PANI-Ag showed distinct morphology from PANI. It is seen in the micrographs that silver (Ag) nanoparticles (affirmed by XRD) are appropriately distributed in the PANI network (Fig. 4(b)). PANI doped with a noble metal, for example, Ag gives great electrical conductivity. It can be assured from SEM micrograph, Ag nanoparticles spots are well attached to the PANI matrix because of the potent attraction of Ag for nitrogen^44^. It was found that Ag nanoparticles functioned as conductive connections between the PANI grid that upgrade the electrical conductivity of the PANI-Ag nanocomposite. Figure 4(c) demonstrates the interaction of PANI-Ag nanocomposite with ferritin which was utilized as an electron transport mediator from profoundly covered redox dynamic site of the enzyme. Figure 4(d) demonstrates the agglomeration of PANI-Ag/Frt/GOx which is utilized.

### Electrochemical investigation of PANI-Ag/Frt/GOx bioanode

**Figure 5:**
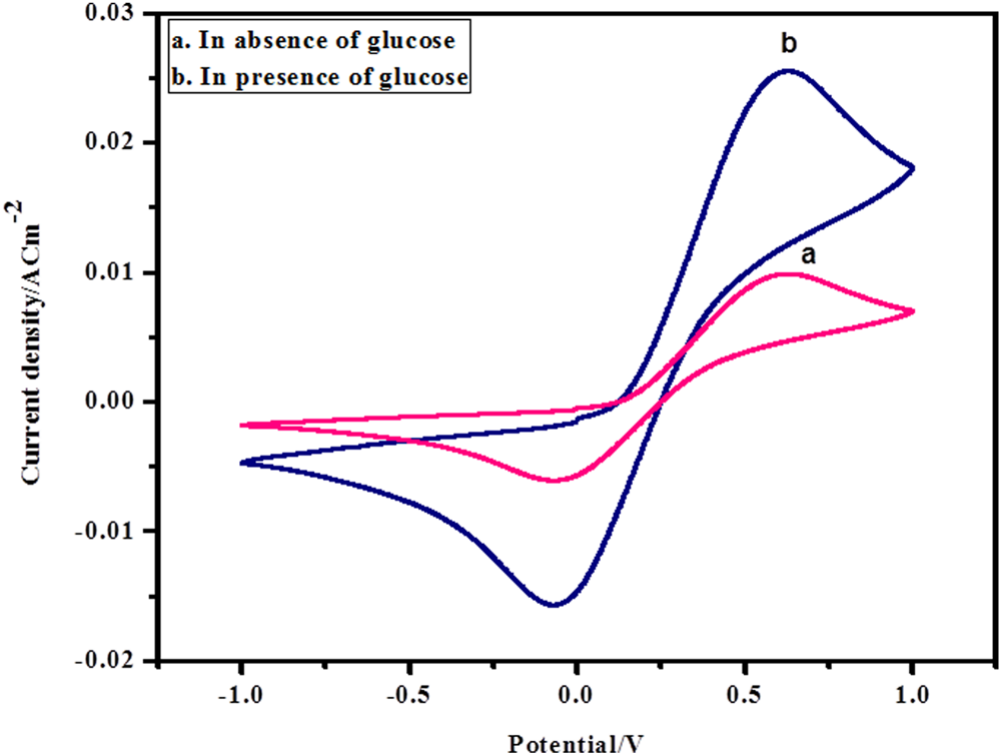
CVs of (**a**) PANI-Ag/Frt/GOx in absence of glucose in PBS of pH 7.0 (**b**) PANI Ag/Frt/GOx in 40 mM glucose in PBS of pH 7.0 at room temperature at a scan rate of 100 mVs^−1^.

To examine the interceded electron transfer by utilizing ferritin as a mediator, the cyclic voltammetry of PANI-Ag/Frt/GOx bioanode was carried out. The nitrogen cleansing was done in every investigation for keeping up the action of the enzyme. The GOx cast on cationic aminated surface of ferritin with successive coverage of glutaraldehyde added the significant improvement of its environmental and thermal stabilities^45^. The life expectancy of adsorbed enzyme over the surface of PANI-Ag/Frt electrode was observed to be 40 days (approx). The manufactured PANI-Ag/Frt/GOx bioanode demonstrated a biocatalytic action for the oxidation of glucose to gluconolactone along with a byproduct (H_2_O_2_) in phosphate buffer solution (PBS) of pH 7.0 at room temperature (25 ± 3 °C) as appeared in Fig. 5. It was seen that the PANI-Ag/Frt/GOx modified bioanode delivered extremely good oxidation current (25.40 ± 2 mA cm^−2^) in 40 mM glucose solution. It is considered because of the transformation of glucose to gluconolactone at a sweep rate of 100 mVs^−1^. Be that as it may, without glucose, the redox pinnacle of mediator was detected as it was. The bioanode PANI-Ag/Frt/GOx showed an arrangement of redox peaks at 0.5 and −0.1 V, separately, which showed the covalently linked GOx to the modified PANI-Ag/Frt bioanode retained its biocatalytic activity^44^.

**Figure 6:**
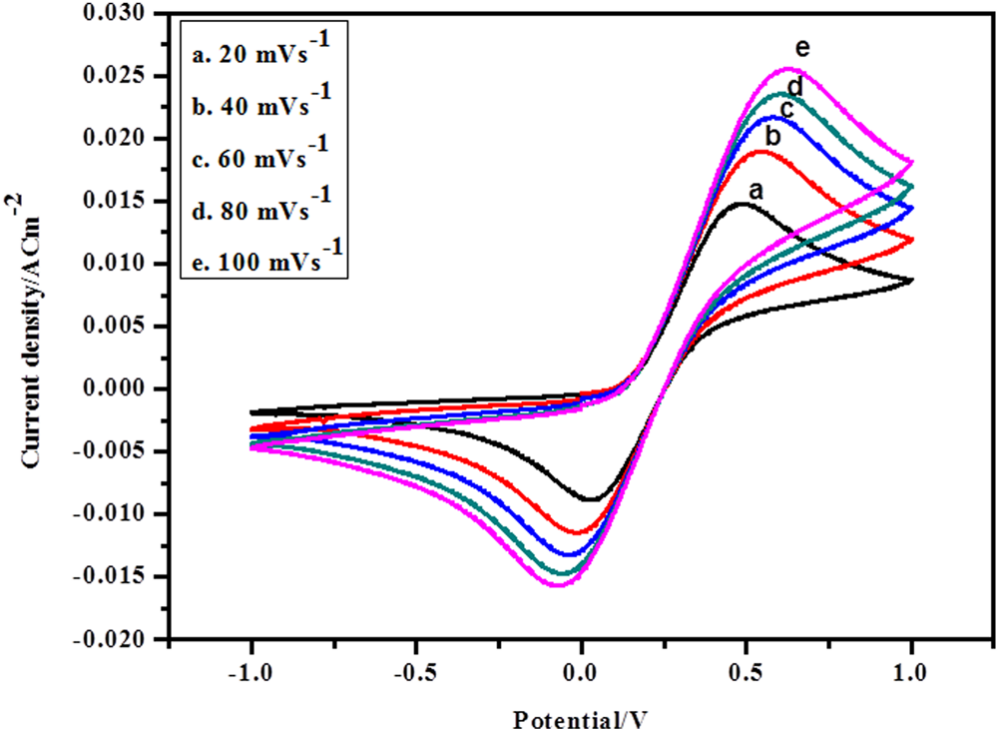
CVs of PANI-Ag/Frt/GOx modified GC electrode in 40 mM glucose in PBS of pH 7.0 at a scan rate (**a**) 20, (**b**) 40, (**c**) 60, (**d**) 80 and (**e**) 100 mVs^−1^.

The critical impact of various scan rates viz, 20, 40, 60, 80 and 100 mVs^−1^ on the catalytic activity is shown in Fig. 6. It is detectable that redox pinnacles of PANI-Ag/Frt/GOx modified bioanode increase directly with the expansion in scan rates. That exhibited the good electrocatalytic nature and quasi-reversible redox behavior of the prepared bioanode. The response of the adsorbed GOx is an average surface controlled phenomenon which is shown by the straight relationship of the pinnacle current with scan rate.

With the assistance of cyclic voltammograms the concentration of the PANI-Ag/Frt/GOx biocomposite on the surface of GC anode was assessed by utilizing Brown–Anson condition^45^:

$${I}_{p}={n}^{2}{F}^{2}{I}^{\ast }Av/4RT$$

where n symbolize the quantities of electrons to be exchanged (in the present case n = 2), F is the Faraday constant (96485 C mol^−1^), I* demonstrates the concentration of the PANI-Ag/Frt/GOx biocomposite on the surface of GC anode (in mol cm^−2^), to be resolved, A is the surface region of the GC electrode (0.07 cm^2^), v shows the sweep rate (100 mV s^−1^), T is the temperature (in Kelvin) and R is the gas constant (8.314 JK^−1^mol^−1^). The surface concentration of the bioelectrode confined by PANI-Ag/Frt/GOx was observed to be 2.21 × 10^−12^ mol cm^−2^.

**Figure 7:**
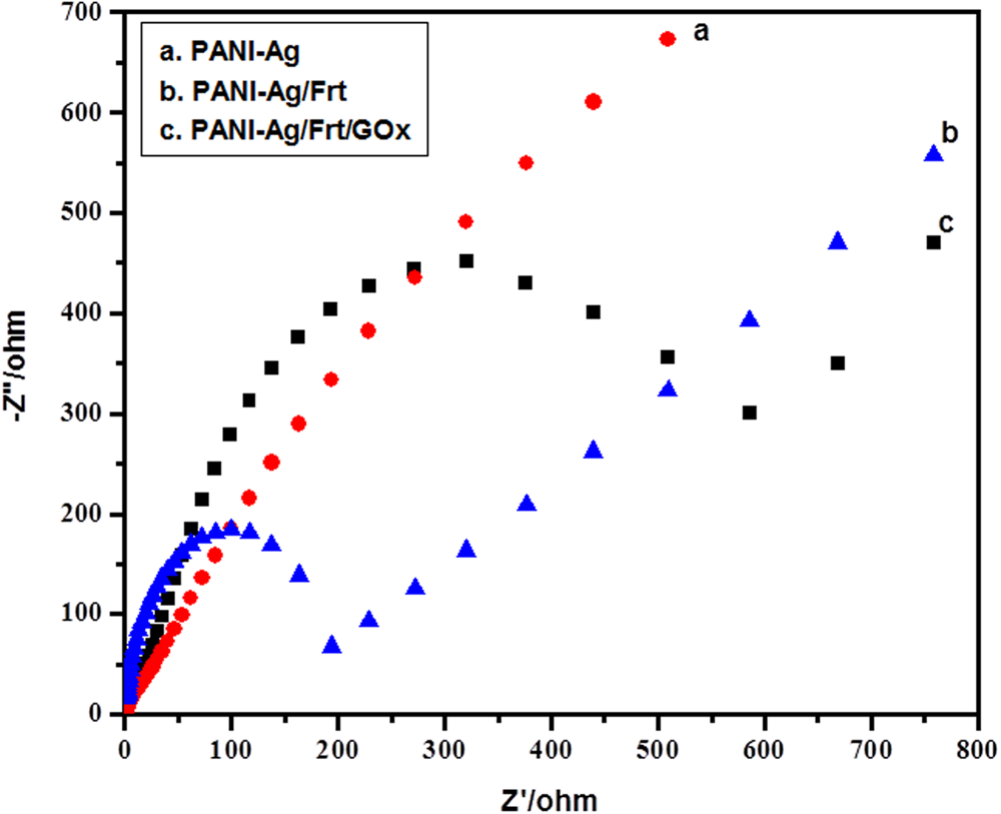
Nyquist plot of PANI-Ag, PANI-Ag/Frt, and PANI-Ag/Frt/GOx modified electrodes in PBS of pH 7.0.

EIS (Electrochemical impedance spectroscopy) is a technique used to know the behavior of electrode material at the interface of electrode-electrolyte. The Fig. 7 shows the Nyquist plot of PANI-Ag, PANI-Ag/Frt, and PANI-Ag/Frt/GOx modified electrodes in PBS of pH 7.0. Generally, the Nyquist plot consists of two part, a straight line and a semi-circle, wherein the straight line indicates the diffusion controlled reaction while the diameter of the semi-circle is suggestive of the resistance to charge transfer (R_ct_)^46^. From the results of EIS, it is evident that the modified electrodes have undergone redox reactions which are driven by the diffusion controlled pathway. In Fig. 7 (a) the slope of the curve towards imaginary component (Z”) is remarkably higher, that indicates the accumulation of significant amount of charge near the electrode surface. On the other hand, the PANI-Ag/Frt bioanode generated a relatively large diameter semi-circle, suggesting the favorable binding of ferritin into the PANI-Ag matrix. Yet, the thousand of metal centers present in ferritin protein, efficiently contribute to the electron transfer, leading to low charge transfer resistance (R_ct_)^46^. The largest semi-circle observed in Fig. 7(c) may be attributed to the successful immobilization of GOx enzyme into PANI-Ag nanocomposite. This implies that the PANI-Ag nanocomposite provides sufficient surface area for the adsorption of GOx resulting in fairly improved bioelectrocatalytic oxidation of glucose.

**Figure 8:**
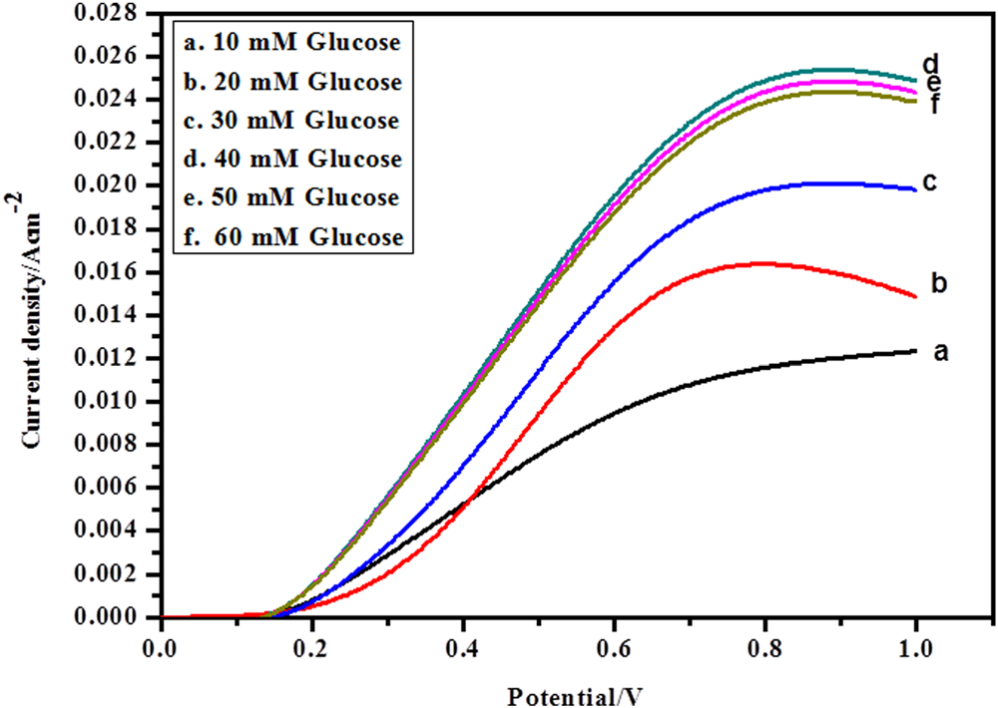
LSVs of PANI-Ag/Frt/GOx modified GC electrode in PBS of pH 7.0 with various glucose concentrations (**a**) 10, (**b**) 20, (**c**) 30, (**d**) 40, (**e**) 50 and (**f**) 60 mM at a scan rate of 100 mVs^−1^.

Linear sweep voltammetry (LSV) was utilized to portray the PANI-Ag/Frt/GOx modified bioanode in presence of various concentrations of glucose i.e. 10, 20, 30, 40, 50 and 60 mM in PBS of pH 7.0 as appeared in Fig. 8. The chart of LSV demonstrates that the electrocatalytic current of modified GC electrode increases directly with the increase of glucose concentration up to 40 mM. After that, any up gradation in current has not appeared. This behavior shows that the reaction satisfies saturation kinetics and the current drops to a steady range that don’t rely on the further hike in glucose concentration.

**Figure 9:**
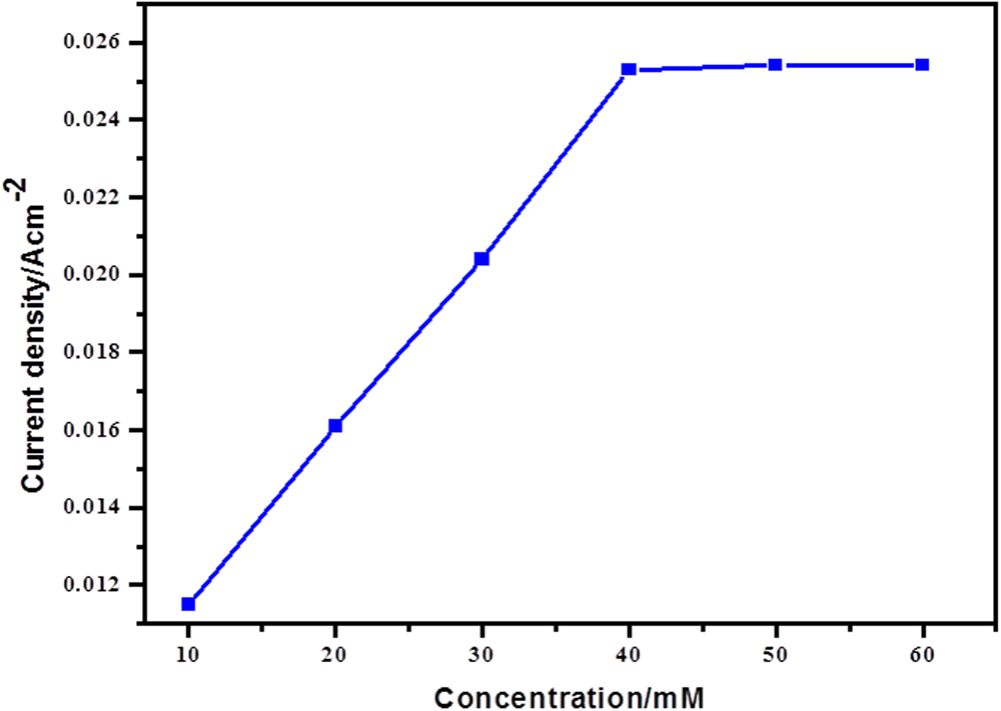
Calibration curves for the glucose concentration vs. oxidation current density.

It is clear that the modified PANI-Ag/Frt/GOx bioanode is dynamic for the catalytic oxidation of glucose by means of the electron exchange mechanism. The calibration curve as a component of glucose concentration versus current density plotted by utilizing this bioanode appears in Fig. 9. It is discernible by the Fig. 9 that current density improves with an increase in the glucose concentration and an immensed current density of 25.4 ± 2 mA cm^−2^ for the oxidation of 40 mM glucose concentration was accomplished at a scan rate of 100 mVs^−1^.

Long term stability of bioanode is a key component in enzymatic biofuel cells. The steadiness of the above PANI-Ag/Frt/GOx bioanode as a component of time (days) was studied. The execution of modified PANI-Ag/Frt/GOx bioanode was studied at 40 mM glucose concentration in PBS of pH 7.0. It was found that following 10 days storage at 4 °C the modified bioelectrode can hold around 88% of its original current density.

## Conclusion

In this examination work conducting polyaniline/silver (PANI-Ag) nanocomposite was synthesized in the presence of silver nitrate precursor. PANI is frequently being used as a conducting polymer with an exceptionally porous nanostructure and outstanding electronic properties for electron transfer. For a better communication between enzymes and the modified bioanode surface polyaniline-silver (PANI-Ag) nanocomposite was used successfully. The electrical conductivity of nanocomposite was improved by fairly conductive silver nanoparticles. PANI-Ag likewise gave a decent support to catalyst immobilization by utilizing a biocompatible arbiter in the middle of, that demonstrates a simplicity for the electron transfer.
